# Modulating Composition and Metabolic Activity of the Gut Microbiota in IBD Patients

**DOI:** 10.3390/ijms17040578

**Published:** 2016-04-19

**Authors:** Mario Matijašić, Tomislav Meštrović, Mihaela Perić, Hana Čipčić Paljetak, Marina Panek, Darija Vranešić Bender, Dina Ljubas Kelečić, Željko Krznarić, Donatella Verbanac

**Affiliations:** 1 Center for Translational and Clinical Research, University of Zagreb School of Medicine, 10000 Zagreb, Croatia; mihaela.peric@mef.hr (M.Pe.); hana.paljetak@mef.hr (H.Č.P.); marina.panek@mef.hr (M.Pa.); donatella.verbanac@mef.hr (D.V.); 2 Clinical Microbiology and Parasitology Unit, Polyclinic "Dr. Zora Profozić", Bosutska 19, 10000 Zagreb, Croatia; tomislav.mestrovic@gmail.com; 3 Department of Internal Medicine, Division of Clinical Nutrition, Clinical Hospital Centre Zagreb, 10000 Zagreb, Croatia; dvranesi@kbc-zagreb.hr (D.V.B.); ljubas.dina@gmail.com (D.L.K.); zeljko.krznaric1@zg.t-com.hr (Ž.K.); 4 Department of Internal Medicine, Division of Gastroenterology and Hepatology, Clinical Hospital Centre Zagreb, 10000 Zagreb, Croatia; 5 Department of Internal Medicine, University of Zagreb School of Medicine, 10000 Zagreb, Croatia

**Keywords:** gut microbiota, inflammatory bowel disease (IBD), dietary interventions, probiotics, fecal microbiota transplantation (FMT), nematodes

## Abstract

The healthy intestine represents a remarkable interface where sterile host tissues come in contact with gut microbiota, in a balanced state of homeostasis. The imbalance of gut homeostasis is associated with the onset of many severe pathological conditions, such as inflammatory bowel disease (IBD), a chronic gastrointestinal disorder increasing in incidence and severely influencing affected individuals. Despite the recent development of next generation sequencing and bioinformatics, the current scientific knowledge of specific triggers and diagnostic markers to improve interventional approaches in IBD is still scarce. In this review we present and discuss currently available and emerging therapeutic options in modulating composition and metabolic activity of gut microbiota in patients affected by IBD. Therapeutic approaches at the microbiota level, such as dietary interventions alone or with probiotics, prebiotics and synbiotics, administration of antibiotics, performing fecal microbiota transplantation (FMT) and the use of nematodes, all represent a promising opportunities towards establishing and maintaining of well-being as well as improving underlying IBD symptoms.

## 1. Introduction

Inflammatory bowel disease (IBD) is a chronic relapsing inflammatory condition which is comprised of two clinically and morphologically different entities: Ulcerative colitis (UC) and Crohn’s disease (CD). Although the etiology of IBD is unknown, the dominant hypothesis suggests the inflammation results from sustained immune response towards altered or pathogenic microbiota within a genetically susceptible host [1].

The human gut microbiota is a diverse microbial community, estimated to contain over 1000 different bacterial species, as well as commensal fungi and viruses [2]. The total number of microbial organisms in human gut is estimated to about 100 trillion, up to ten times the number of cells constituting the human body, while the collective microbial genome, the microbiome, contains approximately a hundred times more genes than the entire human genome [3]. Microbiota substantially increases host metabolic capacity and actively contributes to maintaining gut homeostasis [4].

Recent developments in gene-sequencing technologies and potent bioinformatics tools have enabled new insights into the composition, interactions and effects of microbial communities on human physiology, revealing their role in various pathological conditions [5]. More than 90% of the normal human gut microbiota is composed of species within four major bacterial phyla: *Firmicutes*, *Bacteroidetes*, *Proteobacteria* and *Actinobacteria* [2,6]. Analysis of the microbiome using 16S rRNA sequencing confirmed that microbiota in IBD patients significantly differs from the one present in healthy individuals, with its diversity and bacterial load decreased, and assembling the characteristics of the status known as “dysbiosis” [7,8,9]. More profound studies performed in UC and CD affected patients have shown a clear reduction in *Firmicutes* (especially *Clostridium* groups) and an increase in *Proteobacteria* [6,10,11], with the significant decrease of many protective bacterial species from genera such as *Bacteriodes*, *Eubacterium* and *Lactobacillus* [12,13,14,15]. Although great progress has been made in understanding the role of gut microbiota in IBD, it still remains unknown whether dysbiosis is a cause or consequence of intestinal inflammatory response in IBD.

**Figure 1:**
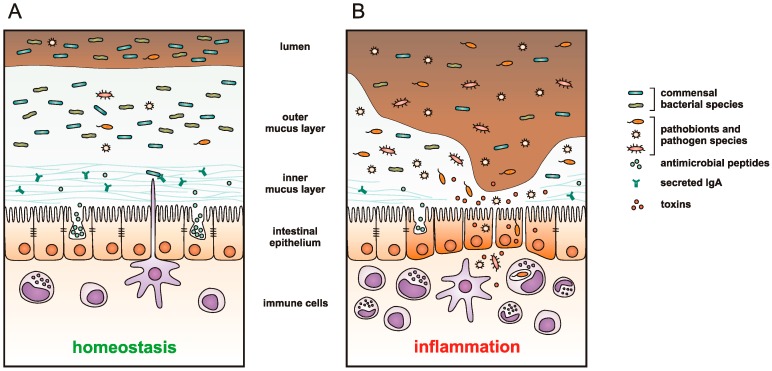
A schematic representation of intestinal mucosa in (**A**) healthy; and (**B**) inflammatory bowel disease (IBD)-affected individual. A thick mucus layer overlies the entire epithelium of the healthy intestine. Bacteria are distributed throughout the outer mucus layer, while the inner layer is thick and resistant to bacterial penetration due to the antimicrobial peptides secreted by the epithelial cells, and immunoglobulin A (IgA) produced by B cells and transcytosed across the epithelial layer. Commensal microbiota suppresses the proliferation of pathobionts (opportunistic bacteria that coexist with commensals in low extent) and enteric pathogens, tuning the host responses towards immunological tolerance and maintaining intestinal homeostasis. Genetic and environmental factors influence intestinal microbiota composition and can lead to mucosal dysbiosis—disruption of commensal microbial community, resulting in outgrowth of pathobionts and colonization of the intestine by pathogen species. These bacterial species produce and secrete toxins, thinning the protective mucus layer, damaging the epithelial layer and, thus, reducing the integrity of the epithelial barrier. The bacteria can gain access to epithelium and mucosal tissue resulting in a strong inflammatory response by the host immune system.

The disturbance of gut microbiota metabolites also contributes to the pathogenesis of IBD. This particularly refers to the decreased production of short-chain fatty acids (SCFA) in IBD-affected patients [16]. SCFA possess a well-documented anti-inflammatory activity and serve as a major source of energy for the gut epithelial cells. Taking all this into account, there is a permanent inflammation process in IBD-affected patients, which can be caused and/or followed by the disruption of gut epithelial cells barrier integrity and presence of the continuous leakage from the gut lumen to the blood (Figure 1).

However, the observed changes in gut microbiota and associated metabolites in IBD patients are not completely consistent among different scientific reports, with the observed discrepancies usually explained by different study design parameters, such as diverse samples (feces/biopsy), sampling location (inflamed/non-inflamed site), disease state (active/non-active), methods of sample analysis, as well as differences in subjects of these studies (age, diet, use of medications) [17]. Thus, there is a dire need for unification and standardization of study designs in order to obtain more consolidated data. Additionally, holistic approaches that take into account different factors able to modify the composition of gut microbiota and manipulate with it, today, represent a powerful tool, which might be very useful for basic and clinical researchers in finding new therapeutic options for patients affected by these devastating diseases.

## 2. Nutritional Patterns and Dietary Interventions

The concept paraphrased as: “You are what you eat” raised by early researchers Brillat-Savarin, Feuerbach and Lindlahr is today as contemporary as ever. Namely, recent changes in dietary trends in the Western world, including dramatic increase in sugar/fat consumption and reduced intake of dietary fibers, fruits and vegetables, have led to specific alterations in structure and function of human intestinal microbiota [18,19], and have been proposed as a major contributing factor to growing incidence of IBD prevalence [20,21,22]. Eating habits are the main significant determinants of the microbial multiplicity of the gut, and dietary components influence both microbial populations and their metabolic activities from the early stages of life. Therefore, effects of short-term and long-term dietary interventions on gut microbiota have been studied in healthy and disease-affected population. De Filippo *et al.* [23] reported that the long-term dietary habits were associated with specific differences in microbial composition. The study compared microbiota from European children fed a typical Western diet, rich in fat, sugar and animal proteins, with that from children in Burkina Faso, fed a traditional rural diet rich in fiber and plant polysaccharides. The results indicated that children from Burkina Faso had increased microbial diversity and richness, together with significantly more *Bacteroidetes* and fewer *Firmicutes* than the European children, with a specific abundance of *Prevotella* and *Xylanbacter*, two bacterial genera absent in microbiota of European children [23]. Another study observed similar trends comparing populations from the US metropolitan areas, rural Malawi, and Amazonas of Venezuela, with clear distinctions in bacterial community signatures and functional gene repertoires between Western and non-Western people, related to the differences in their diet [24]. Interestingly, the microbiomes of US subjects were overrepresented with bacterial genes involved in degradation of glutamine and several other amino acids, catabolism of simple sugars, sugar substitutes and host glycans, thus resembling the carnivorous mammalian microbiomes and reflecting the diet rich in animal proteins, sugar and fat. On the contrary, the microbiomes of the Malawian and Amerindian subjects showed higher representation of glutamate synthase and α-amylase, resembling the microbiomes of herbivorous mammals [24]. To evaluate the impact of short-term dietary interventions on microbiota, David *et al.* [25] performed a five-day controlled feeding study involving an animal-based diet (meat, eggs, dairy) or a plant-based diet (fruits, vegetables, grains and legumes) in healthy subjects The animal-based diet rapidly increased the levels of bile-tolerating bacterial genera such as *Bacteroides* or *Bilophila*, while decreasing levels of plant polysaccharide metabolizing *Firmicutes*. The observed increase in *Bilophila wadsworthia*, a species linked to inducing colon inflammation in mouse models [26], is particularly interesting as it supports the association of Western diet with intestinal microorganisms directly contributing to IBD pathogenesis. Two days after the animal-based diet was stopped, the structure of microbiota retuned to its pre-intervention baseline [25]. Other groups also reported a prompt response of the microbiota to changes in dietary fat or fiber content, with the temporal microbiota community shifts reverting back to its original structure shortly after the intervention was completed [27,28]. In contrast, less than complete microbiota recovery was reported after antibiotic treatment [29,30] or enteric infection [31] throughout the follow-up period.

The Mediterranean diet (MD) is a well-known dietary pattern, attractive for its palatability as well as health benefits. Its characteristic components represent a golden standard of well-balanced nutrition and are recommended by the World Health Organization (WHO) as a prevention of coronary heart disease, certain cancers, and other diseases related to nutritional habits [32]. The influences of MD on IBD have been a topic of investigation by several research groups, and the results confirmed diet’s beneficial effects such as suppression of inflammation and stabilization of intestinal microbiota. Lynnette Ferguson and her team employed transcriptomic technique to test the ability of Mediterranean-inspired diet to reduce inflammation in people with CD [33]. After a six-week diet, a reduction was noted in the recognized biomarkers, C-reactive protein (CRP) and micronuclei numbers, and there was a trend in normalising of the intestinal microbiota—an increase in *Bacteroidetes* and the *Clostridium* clusters, and a decrease in *Bacillaceae* and *Proteobacteria*. Furthermore, mouse models have shown that a diet based on extra virgin olive oil enriched with hydroxytyrosol (components of Mediterranean diet) has the ability to attenuate both the clinical and histological signs of damage, improve disease activity index and reduce mortality in dextran sulphate sodium (DSS)-induced colitis (which is an excellent preclinical system in IBD research) [34]. In addition, natural wine phenolic compounds may act as prebiotics and restore the microbial balance between baleful and protective luminal bacteria, preventing, in turn, inflammatory reactions and reducing mucosal injury in IBD [35]. Yet again, a systematic approach and additional studies are needed to employ such targeted diets and nutritional interventions as a possible IBD therapy.

Over the last few years, researchers started evaluating various dietary approaches and interventions as a part of treatment plan for patients with IBD, both for regulating nutrient deficiencies and influencing disease activity. Exclusive enteral nutrition (EEN) with elemental, semi-elemental or polymeric formulas has been extensively studied, especially in pediatric CD patients [36]. EEN was found as effective as corticosteroids in inducing disease remission in children [37,38,39], was associated with high rates of mucosal healing [40], led to weight gain [41], as well as improved vitamin D, bone health status [42,43], and quality of life after treatment [44]. In addition, several studies have shown that partial enteral nutrition (PEN) can be effective in the maintenance of remission in pediatric patients with CD [45,46,47]. Interestingly, EEN is not a first line therapy for inducing disease remission in adult patients with CD, although it does have benefits for achieving remission in adults with newly diagnosed CD, or in cases when treatment with corticosteroids is not feasible [48,49]. The mechanism by which enteral nutrition is effective for inducing bowel rest in IBD is not fully elucidated. Although several studies reported EEN improves microbiota diversity and increases protective bacterial species [50,51], recent study by Gerasimidis *et al.* [52] found enteral nutrition induced a further decline in diversity, as well as even lower levels of *Faecalibacterium prausnitzii* and *Bacteroides*/*Prevotella* groups in CD patients undergoing EEN therapy. Kaakoush *et al.* [53] also associated EEN with decrease in microbial diversity, hypothesized as depletion of inflammatory commensals found in higher abundance in CD patients, thus resulting in a diminished immune response towards the CD microbiota [54].

The success of enteral nutrition in treating Crohn’s disease has not been transferred to specific whole food dietary approaches, and although the question of what to eat is among the most often asked by patients with IBD, there is currently no specific diet suggested as the best for managing the disease. In an effort to compile available information regarding diet and IBD, Brown *et al.* [55] presented a review of dietary recommendations published by medical organizations in the form of clinical practice guidelines, as well as those listed by patient-centered IBD societies. Although there are substantial variations regarding specific dietary recommendations, most of these guidelines suggest eliminating dairy if lactose intolerant, limiting excess fat, excess carbohydrates and reducing fiber, particularly during disease flares [55].

Recent literature proposed several elimination diets which have become popular with IBD patients. These include the semi-vegetarian diet (SVD), specific carbohydrate diet (SCD), the low-fermentable oligosaccharide, disaccharide, monosaccharide, and polyol (low-FODMAP) diet, IBD-Anti-Inflammatory Diet (IBD-AID) and allergen elimination diet. The SVD allowed milk and eggs, fish once per week and meat once in two weeks, and was rich in dietary fiber. The diet was assessed by a prospective two-year clinical trial and was found highly effective for preventing relapse in CD [56]. The much more restrictive SCD, first described for treating celiac disease [57], was recently popularized in the book “Breaking the Vicious Cycle” with the author using the diet to cure her daughter of UC [58]. The diet restricts the intake of complex carbohydrates and completely eliminates refined sugar, based on a theory that those foodstuffs are poorly absorbed and pass undigested into the colon where they can contribute to dysbiosis and may cause intestinal injury. A retrospective clinical review of pediatric CD patients reported clinical improvement within three months of SCD initiation, and continuing for duration from 5 to 30 months [59]. A prospective pilot study of the SCD also reported beneficial effects of the diet for clinical and mucosal improvement in pediatric patients with CD [60]. The low-fermentable oligo-, di-, mono-saccharides and polyol (FODMAP) diet restricts the intake of foods rich in FODMAPs using a similar premise as SCD; poorly absorbed carbohydrates are rapidly fermented by gut bacteria resulting in bacterial overgrowth [61]. FODMAPs are a large group of dietary sugars (fructose, lactose, fructans, galactans and polyols), found in many common foods such as dairy products, wheat and other grains, fruits and vegetables. Two retrospective pilot studies assessed the low-FODMAP diet for treating IBD and reported an improvement of disease symptoms [62,63]. However, both SCD and low-FODMAP are very restrictive diets and may dramatically reduce overall caloric intake in IBD patients who are already at risk for malnutrition [64]. The anti-inflammatory diet for IBD (IBD-AID) was derived and augmented from SCD by Olendzki *et al.* [65]. IBD-AID limits the intake of certain carbohydrates (refined sugar, lactose, gluten-based grains) believed to stimulate the growth of pro-inflammatory bacteria in the gut, while promoting consumption of anti-inflammatory foods including those with prebiotic and probiotic properties. A small pilot study showed efficacy of the IBD-AID on patients following the diet for 4 weeks, reporting improvement in clinical symptoms and ability to decrease doses of or discontinue the medication treatment [65]. Allergen elimination diet relies on identification and elimination of foods which elicit an IgG antibody response. By measuring IgG4 levels in blood serum after exposure to different food antigens, a pilot study on patients with CD eliminated each patient’s most reactive foods from their diets for four weeks, resulting in significant improvement of disease symptoms [66]. A randomized, double-blind, cross-over diet intervention study reported efficacy of IgG-guided exclusion diet in patients with active CD [67]. The study assessed reactivity of T cells to food antigens *in vitro*, with patients receiving elimination diet for six weeks based on the *in vitro* results, then crossing over to the sham diet. The results indicated significant alleviation of disease symptoms during the IgG diet compared to the sham diet [67].

Although the studies report beneficial results of the aforementioned dietary therapy strategies, these diets lack rigorous scientific evaluation based on clinical trials. However, unlike classical pharmacological trials, dietary intervention trials investigating the effect of food on gastrointestinal disorders are limited due to the lack of placebo control group and other shortcomings like accuracy of capturing information on dietary intake, complex interactions between foodstuffs consumed as well as individual differences in food metabolism [68]. That being said, improving the current methodology of studying dietary interventions is a crucial challenge in developing precise dietary therapy strategies for IBD [21].

Finally, composition and function of microbiota is highly adapted to dietary habits, showing a high level of resilience against short-term perturbations. However, studies also suggest that profound changes in long-term dietary habits, as well as exposure to antibiotics or enteric pathogens, may trigger an abrupt transformation within commensal microbial structure and its function, shifting the intestinal ecosystem into dysbiosis. With this status confirmed in IBD, researchers had a sound rationale for trying to optimize the composition of the altered microbiota in IBD patients not only using long term dietary intervention, but also with the approaches which are able to produce positive therapeutic response more rapidly. Interventions of this type include supplementation with probiotics, prebiotics and synbiotics, the use of antibiotics, as well as more recently introduced therapeutic approaches, like fecal microbiota transplantation (FMT) and treatments with helminths ova.

## 3. Supplementation with Probiotics, Prebiotics and Synbiotics

For more than 15 years researchers are looking for the best definition for pro-, pre- and synbiotics, as well as the unified name for these three terms [69]. When it comes to modulating gut microbiota, these components are considered as one of the key elements in therapeutic approaches toward relieving the symptoms of gastrointestinal disorders and restoring the health.

Probiotics are defined as live microorganisms, which when administered in adequate amounts confer health benefit on the host [70]. A number of reports suggested a beneficial activity of specific bacterial species originally derived from food (dairy products in particular), with probiotics exerting antimicrobial activity, competing with pathogenic microbes and suppressing their growth, improving the host intestinal barrier activity and modulating of the inflammatory response in IBD patients [7,71].

The most evaluated single probiotic strain is *Escherichia coli* (*E. coli*,) Nissle 1917, a non-pathogenic *E. coli*, with randomized control trials showing the strain to be as effective and safe as mesalazine for maintenance of remission in UC patients [72,73]. *Lactobacillus GG* was also found effective for maintenance of remission in UC patients [74], but not in CD patients [75,76]. Other commonly cited probiotic single strains for the treatment of IBD are those of lactobacilli [77], bifidobacteria [78], and the yeast strain *Saccharomyces boulardii* [79]. The most convincing data for using probiotics in IBD comes from studies on commercial probiotic supplement VSL#3, a highly concentrated (450 billion bacteria/sachet) freeze-dried cocktail containing eight different bacterial species: one of the *Streptococcus* genus (*S. thermophilus*), three of the *Bifidobacteria* genus (*B. breve*, *B. infantis* and *B. longum*), and four of the *Lactobacillus* genus (*L. acidophilus*, *L. casei*, *L. bulgaricus* and *L. plantarum*). VSL#3 probiotic cocktail was found beneficial in maintaining clinical remission in UC patients intolerant to salycilates [80] and as well as in inducing remission in patients with active UC receiving conventional therapy [81,82]. A recently published meta-analysis assessed the data from RCTs and concluded the VSL#3 cocktail, given in combination with standard UC therapy, results in higher rates of response and remission when compared to standard therapy alone [83]. VSL#3 was also found effective in maintenance of chronic pouchitis and in prevention of pouchitis after ileal pouch anal anastomosis [84]. Further results from several randomized control trials proved the role of VSL#3 in the management of pouchitis [85], which ultimately led to European Crohn’s and Colitis Organisation (ECCO) guidelines suggesting the use of this particular probiotic mixture both for maintenance of antibiotic-induced remission and for prevention of pouchitis [86].

On the other hand, although beneficial effects of probiotics have been established in treating gut disorders, currently there is insufficient data demonstrating their impact on the host microbiota. One of the few studies published on this topic reported that VSL#3 induced an increase in concentration of *Lactobacillus*, *Bifidobacterium* and *Streptococcus salivarius* in the gut, which however returned to the basal levels 15 days after the treatment [80]. Recently, researchers have taken probiotics to the next level, genetically modifying bacterial species to produce specific immunosuppressive mediators, *i.e.*, IL-10 [87,88] and antioxidant enzymes, *i.e.*, superoxide dismutase—SOD [88,89,90]. This is an interesting approach for mucosal delivery of selected proteins and could prove a feasible strategy for downregulating gut inflammation in IBD.

With only a few clinical reports describing the same study design, probiotic strains and doses, it is difficult to compare the results and gain definite conclusions for or against probiotics in IBD therapy. However, the evidence strongly supports using probiotics as supplements for the conventional therapy of UC patients. *E. coli* Nissle 1917 and VSL#3 both received a very strong “A” recommendations in American Recommendations for probiotic use for the maintenance of remission in UC [91], and ECCO guidelines also recognized the role of VSL#3 probiotic mixture for treating relapsing, mild-to-moderate UC [92]. The reports on the use of probiotics in CD do not suggest any disease improvement, so probiotics are not advocated for this patient population [93].

Prebiotics are food ingredients, usually non-digestible carbohydrates, which can selectively stimulate growth and promote activity of protective commensal microorganisms thus providing benefits upon host well-being and health [94]. Prebiotics are metabolized by anaerobic microorganisms, producing short-chain fatty acids (SCFA) and gas. Elevated levels of SCFA, particularly butyrate acids, can lower the pH of the colon and promote the growth of lactobacilli and bifidobacteria, while decreasing potentially pathogenic bacteria [95]. Moreover, butyrate improves the epithelial barrier function in IBD and exerts anti-inflammatory action via suppression of NFκB pathway [96].

There are very few clinical studies reporting prebiotic therapy in IBD. Germinated barley foodstuff (GBF) is one of the most frequently investigated prebiotics. Rich in glutamine and hemicellulose, GBF was found to significantly improve clinical activity scores in patients with mild-to-moderate UC, increasing *Bifidobacterium* and *Eubacterium* levels and elevating the concentration of fecal butyrate [97]. Supplemented for maintenance of remission in UC patients, GBF provided lower relapse rates than conventional therapy alone [98], lowering the serum levels of proinflammatory cytokines interleukin 6 (IL-6) and IL-8 [99], and CRP [100]. Other studies investigated the effect of fructo-oligosaccharides (FOS) in CD patients and found reduced disease activity index and increased mucosal bifidobacteria [101]. However, the subsequent randomized control trial did not confirm these findings reporting no significant improvement in CD patients receiving FOS [102]. A small randomized control trial showed that oligofructose-enriched inulin supplemented mesalazine therapy in mild-to-moderate active UC displayed a significantly lower level of fecal calprotectin compared to placebo group, indicating reduction in inflammation [103]. In cases of pouchitis, inulin supplementation was associated with increased level of butyrate, lower concentration of *Bacteroides fragilis* and a decreased endoscopic inflammation [104].

These studies, although showing a certain degree of prebiotic efficacy in IBD, display very heterogeneous sample sizes and study designs. Therefore, the guidelines are very cautious in identifying the role of prebiotics in IBD and do not advocate the use of prebiotics in either UC or CD [92]. However, the solution is pursued in using combination of the mutually supportive pro- and prebiotics in the form of synbiotics.

Synbiotics consist of dietary supplements combining prebiotics and probiotics with a synergistic beneficial effect on host health [105]. Synbiotic combinations present a promising opportunity for clinical investigation of their benefit and potential use in IBD; however a very few studies supporting the usefulness of symbiotic supplementation were published.

The most common synbiotic combinations available include bifidobacteria and fructooligosaccharides (FOS), *Lactobacillus GG* and inulins, and bifidobacteria and lactobacilli with FOS or inulins. Combination of *Bifidobacterium longum* and inulin-oligofructose was evaluated in the randomized control pilot trial as a supplement to conventional therapy of patients with active UC, showing a beneficial effect in improving clinical activity index compared to placebo [106]. Another randomized control trial showed synergistic effect of a combination of *B. longum* and psyllium compared to the probiotic or prebiotic alone, in supplemental therapy of UC patients [107]. Supplementing anti-inflammatory therapy with a *B. breve* Yakult strain and galacto-oligosaccharide had a significant effect on improving endoscopic score in mild-to-moderate UC [108].

Although benefits associated with prebiotics and probiotics are favorable, researchers are cautious about drawing firm conclusions because benefits vary, depending on the type and the amount of pre- and probiotic consumed. Therefore, more human studies need to be done to provide a better understanding of their direct effect on health.

## 4. Use of Antibiotics

The role of antibiotics in the treatment of septic complications of the IBD (namely wound infections and abscesses) is well-established; on the other hand, their common usage and benefits in the treatment of the primary disease processes of CD, UC and pouchitis have thus far not been fully supported by carefully-designed clinical trials [109,110]. Nevertheless, the rationale for antimicrobial therapy in IBD stems from a large body of evidence that highlights luminal bacteria (and perhaps viruses and fungi) as important players in the pathogenesis of IBD [7,11]. It has been shown that IBD is characterized by a higher microbial load in general and a bigger prevalence of harmful bacterial genera, such as *Escherichia* or *Shigella*, than protective ones, such as *Faecalibacterium* [111].

Therefore antibiotic therapy has the potential to influence the course of IBD by decreasing the total gut concentrations of bacteria and fungi, modifying the composition of the luminal microbiota in favour of beneficial bacteria, as well as decreasing bacterial tissue invasion and formation of microabscesses [112]. By using high-throughput sequencing, Dethlefsen *et al.* [113] demonstrated that ciprofloxacin usage can result in rapid reduction of gut microbiota diversity with substantial effects on roughly one third of bacterial taxa. Combined use of metronidazole and ciprofloxacin in patients with IBD can promptly deplete the concentration of intestinal microbiota even on the first day of therapy, although there is an associated rebound effect with subsequent rise in mucosal bacteria one week after the intervention, providing a rationale for prolonged antibiotic usage [114,115]. The latter can be achieved with rifaximin due to its high safety profile and potential for long-term treatment. The study on CD patients showed that rifaximin has the propensity to increase concentrations of beneficial bacterial constituents such as *Atopobium*, *Bifidobacterium* and *Faecalibacterium prausnitzii*, but its long-term impact on gut microbiota has yet to be established [116].

Metronidazole is the most investigated antimicrobial drug in the treatment of CD, although the majority of studies are observational or without adequate statistical power. In one of the first placebo-controlled, double-blind trials, no difference between metrondiazole and placebo-treated patients was observed, but there was a positive trend in favour of metronidazole in individuals with the disease affecting only the colon [117]. In the multicentre National Cooperative Swedish study, metronidazole showed similar effects as sulfasalazine in the primary treatment of CD [118], while Sutherland *et al.* [119] demonstrated effectiveness of mentronidazole in decreasing the Crohn’s Disease Activity Index. Patients using ciprofloxacin for 10 weeks in the treatment of perianal fistulising CD showed bigger percentage of fistula remission [120], which is a finding supported by a recent meta-analysis [121], while patients with active CD on rifaximin therapy had a better clinical outcome compared to patients who received only placebo [122]. Both metronidazole and ciprofloxacin have been used as an adjuvant treatment of bacterial overgrowth in order to decrease bacterial translocation and reduce disease severity [123].

Trials of antibacterial agents for UC are scarce with controversial results, albeit antibiotic adjuvant therapy is often pursued in severe forms of the disease. Oral vancomycin was tested in a double-blind controlled trial as a therapeutic approach in acute exacerbations of idiopathic colitis, but no significant difference has been found between the two treatment groups (with only a slight trend towards a reduction of surgery in the treatment group) [124]. In patients with severe UC, intravenous metronidazole administered together with corticosteroids was not superior to a placebo [125], while in different study rifaximin resulted in a significant improvement of rectal bleeding, stool frequency and sigmoidoscopic score [126]. Studies on ciprofloxacin show different results with mostly nonsignificant trends, but lower treatment-failure rates are sometimes observed [127]. A recent retrospective, multicentre study used an oral wide-spectrum antibiotic cocktail in children with moderate-severe refractory UC (amoxicillin, doxycycline, metronidazole, vancomycin), resulting in complete clinical remission for 47% of the included children without additional interventions [128].

In short, antibiotics truly constitute a viable supplementary treatment option for reducing luminal bacterial load, risk of progression and relapse, as well as disease severity [129]. Animal models of IBD suggest that not all bacterial species are created equal when it comes to inducing inflammation of the gastrointestinal tract [130,131]; hence it is not reasonable to expect that all antibiotic regimens will be similarly effective in all groups of patients. Even though a majority of studies focused on metronidazole and ciprofloxacin, their side effects have to be taken into account, which means there is definitely a need for both better-designed clinical studies and novel antimicrobial agents.

## 5. Fecal Microbiota Transplantation

Fecal microbiota transplantation (FMT) is a procedure of preparing a fecal suspension from a healthy donor and introducing it into the gastrointestinal tract of a diseased individual, in order to restore recipient’s gut microbiota [132]. This method is becoming increasingly accepted as an effective and safe intervention in some gastrointestinal (GI) diseases, likely due to the restoration of a disrupted microbiome [133]. FMT was first noted in the 4th century by traditional Chinese medicine as the procedure of administrating human fecal suspension by mouth for treating patients suffering from food poisoning or severe diarrhea, and as such was used for centuries. For example, the evidence exists that similar approach was applied in 16th century for the treatment of abdominal diseases accompanied with severe diarrhea, fever, pain, vomiting and constipation [134]. During the World War II, German soldiers reported that Bedouin of Northern Africa ingested fresh, warm camel dung as a treatment for dysentery [135].

The first report of FMT in the modern literature was for the treatment of pseudomembranous colitis in 1958 [136]. The authors used fecal retention enemas to completely cure four patients with refractory disease not responsive to antibiotics. In 1981, sixteen patients with the same diagnosis were successfully treated using the fecal retention enema technique [137]. More recently, FMT spurred a lot of interest due to its positive effects in managing *Clostridium difficile* infection (CDI). CDI is a condition of GI microbiota imbalance, characterized by *C. difficile* overgrowth, with the disease traditionally managed with a course of different antibiotics. However, it has been shown that antibiotic therapy does not alleviate the problem but rather results with exacerbated microbiota dysbiosis and approximately 20% of patients develop recurrent disease [138]. FMT was first proposed as a CDI therapy in 1983 [139] and was soon found to be an ideal treatment for the disease, restoring the balance of intestinal microbiota in stable and durable manner [140]. One of the systematic CDI literature reviews encompassing 317 patients treated across 27 case series and reports showed disease resolution in 92% of cases with no adverse effects [141]. Moreover, a randomized study which compared FMT and traditional antibiotic treatment for recurrent CDI, showed a resolution of disease observed in 93% and 31% patients, respectively [142]. This evidence turned the tide in favor of the FMT which ultimately resulted in American College of Gastroenterology endorsing FMT as therapy in their Guidelines for Managing Relapsing CDI [143]. Additionally, in 2014. The European Society of Clinical Microbiology and Infectious Diseases (ESCMID) revised European CDI Treatment Guidelines to strongly recommend FMT as therapy for patients with multiple recurrent episodes of CDI [144].

The success of FMT in treating CDI has raised the possibility that FMT could be beneficial in other conditions associated with gut microbiota dysbiosis, such as inflammatory bowel disease (IBD). The first report on using FMT for managing IBD was published in 1989, with the author treating his ulcerative colitis (UC) by self-administering a fecal retention enema from a healthy donor [145]. The disease, which was active for seven years and refractory to steroids and salicylates, turned symptom-free in only six months after the procedure with no signs of acute inflammation in colonic mucosa. A case series of six UC patients followed, which all achieved disease remission after FMT with no relapse in 13 years follow up [146,147]. The latest systematic review and meta-analysis of 18 studies including 122 patients with IBD treated using FMT reported a disease remission rate of 45%, with subgroup analyses demonstrating a pooled estimate of clinical remission of 22% for UC and 60.5% for CD [148]. The studies included in the review varied in disease type, phenotype and severity, lacked uniformity in treatment protocols and delivery route, and did not include control groups, making it difficult to draw firm conclusions on the safety and efficacy of the FMT procedure in IBD. The studies, however, do suggest that FMT is not as effective in treating IBD as it is in treating CDI.

In contrast to CDI, which is a direct result of disrupted gut microbiota by antibiotics, IBD is a far more complex pathological condition with genetic, immunological and environmental factors contributing to microbiota disbalance, and it is still not clear if the dysbiosis in IBD is a cause or an effect of the inflammatory process. Also, although most of the studies did not associate FMT with any serious adverse events, some safety concerns have been raised due to reports of FMT inducing disease flares in some IBD patients [149,150]. Two randomized control trials investigating FMT in IBD patients were recently published [151,152]. The first study recruited 75 patients with active UC and administered weekly FMT or water enema for six weeks. The results showed broader microbiota diversity in the FMT group compared to control group, and a statistically significant difference in disease remission between the groups of 24% and 5%, respectively [151]. The second study recruited 50 patients with mild or moderate active UC and provided two FMT treatments, one at the start of the study and the other three weeks later. This study however did not result in a statistically significant difference in remission between the FMT and control groups [152]. Currently, there are 21 registered open studies on FMT in IBD listed on clinicaltrials.gov: nine on UC, six on CD and six on IBD in general. These ongoing trials will generate even larger body of data in the following years and give more insight on the role of FMT in the management of IBD.

Our understanding of the human microbiome has progressed exponentially over the last years. After identifying bacterial species which constitute the microbiota, we can now associate them with various pathological changes in the gut. FMT initiated a revolution in treating microbiota dysbiosis, as it was found very effective for curing CDI and may be a promising therapy in other gastrointestinal diseases, with studies investigating the efficacy of FMT for extra-intestinal disorders associated with gut microbiota, *i.e.*, metabolic diseases, neuropsychiatric disorders, autoimmune diseases, allergic disorders, and tumors. However, the main drawbacks to the current fecal bacteriotherapy are twofold: (i) incomplete characterization of the material delivered into the patient; and (ii) invasive administration route. Thus, the future of the FMT is in defining the beneficial microbial communities as “active ingredients”, establishing well-characterized cultures to be used as next generation therapeutics [153,154], as well as developing oral formulations to remove the need for invasive gastrointestinal delivery procedures [155,156].

## 6. Investigational Approaches

One theory that gained popularity over time is that increased incidence of diseases such as CD and UC, but also a plethora of other autoimmune disorders in the developed world can be attributed to the improved sanitation and reduced human exposure to childhood pathogens—a theory also known as “hygiene hypothesis” [157,158]. Parasitic helminths have coevolved with the immune system of mammals over many millennia with a principal aim to survive in the host [159]. In order to promote their own survival, they have become remarkably efficient modulators of host immunological processes, forming a tolerant environment with suppressed inflammation [157].

Based on the increasing prevalence of IBD which was reversely correlated to the prevalence of helminths in the United States, Joel Weinstock proposed the “inflammatory bowel disease hygiene hypothesis” [160]. It is hypothesized that exposure to helminths might provide protection against CD, UC and other forms of bowel inflammation. Such claims were substantiated by a study conducted in sub-Saharan Africa where intestinal infestation with helminths is abundant among the inhabitants [161]. Low incidence and prevalence of IBD in that population could not have been explained solely by genetic factors, as the incidence in black population of the USA and Great Britain was akin to the Caucasian population in those countries [162]. Although his hypothesis was purely observational and based on correlation, it prompted further research of this phenomenon.

Thus, in recent years various attempts have been made to exploit the hygiene hypothesis in the treatment of IBD via the controlled re-introduction of parasitic helminths [162,163,164,165]. Two groups of gastrointestinal parasitic nematodes have been predominantly used in studies on humans—whipworms (*Trichuris* species) and hookworms (*Necator americanus*). This alternative therapeutic model is based on a reasonable suggestion that the biocenotic relationship between the host and these parasites represents a mutualism rather than a typical parasitism [157]. In addition, the fact that parasitic helminths and the commensal flora share their environmental niche [166], helminth-microbiota interactions also play an important role in the development of IBD [163].

Whipworms are one of major groups of soil-transmitted helminths with a simple and direct life cycle [167]. Infection occurs after the ingestion of embryonated eggs, which are the source of infective larvae that develop to adult worms in the large intestine, partially implanting themselves within the epithelial lining. In order to interrogate IBD hygiene hypothesis in clinical trials on humans, *Trichuris suis* (*T. suis*) or pig whipworm was selected since it met all the obligatory safety prerequisites [162]. *T. suis* is species-specific for pigs, which represent its natural host; nevertheless, this nematode can briefly colonize human gut without causing any symptoms (in contrast to *Trichuris trichiura* or a human whipworm) [168]. After ova of *T. suis* are ingested, the eggs hatch and the worms can be found in the human intestine for several weeks, which means that treatments need to be repeated at specific intervals [161]. Still, such absence of chronic infection is advantageous as it removes any broader public health issues [169].

Inaugural open-label trial was studying effects of the application of live *T. suis* ova in four patients with CD and three patients with UC, each receiving a single dose of 2500 viable eggs [170]. This study demonstrated alleviation of symptoms in treated patients without any overt side-effects. A second attempt was also an open-label trial, which tested repeated dosing in 29 patients with active CD [171]. All participants ingested 2500 viable eggs every three weeks for a total of 24 weeks. At week 12, 66% of the test subjects achieved clinical remission. A randomized, double blind, placebo-controlled trial of *T. suis* in 54 adult patients with active UC was published in the same year [172]. In this study, participants received either 2500 viable *T. suis* eggs or a placebo orally at two-week intervals for 12 weeks, which resulted in a significant improvement in patients receiving the agent when compared to the control group. Recently, Sandborn *et al.* [173] conducted a multi-center, randomized, double-blind, placebo-controlled trial designed to assess the safety and tolerability of single escalating oral doses of up to 7500 viable ova in patients with CD, not noting any short term (2 weeks) or long term (6 months) adverse effects. However, preliminary results of two larger studies delivered somewhat discouraging results. In TRUST-1 and FALK trials, 250 US patients and 240 European patients with CD, respectively, did not show any significant improvement in disease activity index or higher remission rates in diseased individuals [169]. In fact, the study on European patients was even discontinued due to the monitoring committee’s recommendation on its lack of efficacy [169]. Albeit such findings of *T. suis* use in CD patients are not encouraging, results from several ongoing trials in patients with UC are still fervently awaited.

Hookworms and their utilization in the treatment of IBD is slightly more provocative than using *T. suis*, as they are true pathogens associated with substantial morbidity throughout the world [174]. The life cycle of *Necator americanus* (*N. americanus*), the most widely distributed human hookworm, begins with eggs being shed in the feces of infected individuals [175]. The larvae that hatch from embryonated eggs infect the human host by penetrating the skin, subsequently traveling via the circulation and lungs to reach the small intestine where they can attach to the intestinal mucosa and cause serious anemia [174,175]. Still, experimental infections with small number of *N. americanus* larvae are safe and generally well tolerated; furthermore, chronic nature of hookworm infection provides additional benefits, as there is no need for continuous administration of the parasite [163].

A pilot study from Croese *et al.* [176] has established a potential for *N. americanus* as a candidate parasite in treating individuals with IBD. In this study, nine participants with CD (five with active disease and four in remission) were percutaneously inoculated with 25 to 100 *N. americanus* larvae. Eighty percent of the participants with active disease had shown improvement in their illness, and the same percentage achieved remission. Other research has shown that the immune response to hookworm infection can be compared to other intestinal helminths (including whipworms), with the expansion of regulatory T cells (T reg), increased expression of IL-10 and transforming growth factor β (TGFβ), as well as reductions in IL-23, IL-17 A and interferon γ (IFNγ) levels [163,177,178].

Recent studies have shown that human infection with hookworms is associated with an increase in both richness and diversity of the gut microbiota [179], which is in turn linked to a “healthier” and more functional intestinal homeostasis [163,180]. The most recent study exploring the effect of experimental infection with *N. americanus* on the human intestinal microbiota did not show a major impact of acute hookworm infection on the community structure of gut microbiota, although a minor increase in bacterial richness has been observed [181]. In order to examine this hypothesis in greater detail and to move a step forward towards an actual therapeutic use, larger trials on humans in different inflammatory disease settings (with the examination of both fecal and mucosally-associated bacterial communities) are necessary.

## 7. Conclusions and Future Perspectives

A state of unbalanced gut microbiota (dysbiosis) and defects in innate and adaptive immune responses have been repeatedly recognized as key stakeholders in the development of gut inflammatory processes leading to IBD. Technological breakthroughs that now enable a more comprehensive characterization of this complex microbial eco-system, together with the increasing number of studies that demonstrate the impact of diet on gut microbiota, provide a robust rationale for further exploration of the link between the gut microbiota, the diet, and the development of IBD.

Aside from the diet that can influence microbial community composition both in the short and long term, this review has summarized an array of dietary and associated strategies available for modulating either the composition, or immunometabolic activity of the gut microbiota, in order to treat or halt the progression of IBD—most notably supplementation with probiotics, prebiotics and synbiotics. Additionally, the review reports recently established new approaches such as FMT and potential to use nematodes as therapeutic tool in IBD. Applying FMT as a treatment modality in IBD is still in its infancy, thus more research needs to be carried out in order to elucidate its ultimate utility. Controlled re-introduction of parasitic helminths represents a viable (albeit unconventional) approach; however, additional larger and thorough studies are needed for a final verdict.

In any case, as our understanding of all the underlying mechanisms and contribution of the gut microbiota to IBD is improving, novel strategies and therapeutics to modulate the microbiota for the treatment and prevention of UC and CD are on the horizon. Moreover, it may be possible to use the knowledge of microbial communities and their metabolic activities to detect potential IBD marker before conventional diagnostics can. All these insights could be utilized in the future to stratify individuals more precisely, consequently apply more efficient nutritional and treatment interventions and thus make a major step forward towards a true personalized medicine.

## Abbreviations

IBD	inflammatory bowel disease
CD	Crohn’s disease
UC	ulcerative colitis
FMT	fecal microbiota transplantation
SCFA	short-chain fatty acids
CRP	C-reactive protein
EEN	exclusive enteral nutrition
PEN	partial enteral nutrition
SVD	semi-vegetarian diet
SCD	specific carbohydrate diet
low-FODMAP	low-fermentable oligosaccharide, disaccharide, monosaccharide, and polyol diet
IBD-AID	IBD-anti-inflammatory diet
ECCO	European Crohn's and Colitis Organisation
GBF	germinated barley foodstuff
CDI	*Clostridium difficile* infection
ESCMID	European Society of Clinical Microbiology and Infectious Diseases
